# Reconstruction of a genome-scale metabolic model and in-silico flux analysis of *Aspergillus tubingensis*: a non-mycotoxinogenic citric acid-producing fungus

**DOI:** 10.1186/s13068-024-02506-4

**Published:** 2024-05-28

**Authors:** Mehak Kaushal, Daniel J. Upton, Jai K. Gupta, A. Jamie Wood, Shireesh Srivastava

**Affiliations:** 1 Systems Biology for Biofuel Group, International Centre for Genetic Engineering and Biotechnology, ICGEB Campus, Aruna Asaf Ali Marg, New Delhi, 110067 India; 2 Department of Biology, University of York, Wentworth Way, York, YO10 5DD UK; 3 Department of Mathematics, University of York, York, YO10 5DD UK; 4 Present Address: Perfect Day India Pvt. Ltd., Bangalore, India; 5 Present Address: JKG: Zero Cow Factory, Surat, India

**Keywords:** *Aspergillus tubingensis*, Lignocellulosic biomass, Biomass composition, C-sources, Genome scale metabolic model, Flux balance analysis, Citric acid

## Abstract

**Background:**

*Aspergillus tubingensis* is a citric acid-producing fungus that can utilize sugars in hydrolysate of lignocellulosic biomass such as sugarcane bagasse and, unlike *A. niger*, does not produce mycotoxins. To date, no attempt has been made to model its metabolism at genome scale.

**Results:**

Here, we utilized the whole-genome sequence (34.96 Mb length) and the measured biomass composition to reconstruct a genome-scale metabolic model (GSMM) of *A. tubingensis* DJU120 strain. The model, named *i*MK1652, consists of 1652 genes, 1657 metabolites and 2039 reactions distributed over four cellular compartments. The model has been extensively curated manually. This included removal of dead-end metabolites and generic reactions, addition of secondary metabolite pathways and several transporters. Several mycotoxin synthesis pathways were either absent or incomplete in the genome, providing a genomic basis for the non-toxinogenic nature of this species. The model was further refined based on the experimental phenotypic microarray (Biolog) data. The model closely captured DJU120 fermentative data on glucose, xylose, and phosphate consumption, as well as citric acid and biomass production, showing its applicability to capture citric acid fermentation of lignocellulosic biomass hydrolysate.

**Conclusions:**

The model offers a framework to conduct metabolic systems biology investigations and can act as a scaffold for integrative modelling of *A. tubingensis*.

**Supplementary Information:**

The online version contains supplementary material available at 10.1186/s13068-024-02506-4.

## Background

*Aspergillus* is a diverse genus, present ubiquitously and of immense medical and economical importance [1]. The *A. niger* clade is an industrial workhorse for production of diverse products such as enzymes (α-amylases, lipases, cellulases, pectinases), bulk chemicals (citric acid, gluconic acid) and secondary metabolites that show antioxidant, anticancer or antibiotic properties (asperazine or Naphtho-Gamma-Pyrones (NGPs)) [2]. However, they also produce secondary metabolites (mycotoxins) such as ochratoxin A (OTA) and fumonisin B_2_ which may pose a health threat in the food and feed chain [2]. According to a previous study on 69 industrial strains of *A. niger*, 83% were found to produce fumonisin B_2_, 33% could produce OTA and 26% produced both mycotoxins [3]. It is a serious concern that the three strains (NRRL 337, NRRL 3112, and NRRL 3122) which are most widely used in industry for citric acid production tested positive for both mycotoxins. Similarly, the study highlighted that the most popular *A. niger* strains used in academic research (NRRL 3 (92 papers), NRRL 2270 (75 papers), NRRL 599 (31 papers)) were fumonisin producing [3]. Strains capable of producing these toxins were found to be producing these on the media recommended for citric acid production as well and the toxins were detected in the fungal biomass making the leftover biomass unsuitable for animal feed. The study also found that the strains identified as *A. acidus* (42 strains), *A. brasiliensis* (18 strains) and *A. tubingensis* (83 strains) were non-toxinogenic [3]. Thus, biomass of these strains could potentially be used as feed to create added value. *A. tubingensis*, in particular, is already an established cell factory for production of various enzymes including glucose oxidase, phytase and xylanase, and bulk chemicals such as citric acid and ascorbic acid. *A. tubingensis* G131 strain has shown promising results for production of various NGPs (such as fonsecin, fonsecinone A and fonsecin B) which have potential application in the food and cosmetic industries [4]. *A. tubingensis* strains capable of degrading waste such as polyester polyurethane (PU) and a serious mycotoxin, Ochratoxin A (OTA), have also been identified recently [5, 6].

After the advent of whole genome sequencing, it became easier to identify putative genes involved in the biosynthesis of secondary metabolites. Comparative studies on genome sequences from the same species or genus help in identifying biosynthetic pathways, ultimately indicating an organism’s capability to produce a specific type of secondary metabolite. For example, presence of a gene cluster for OTA biosynthesis in *A. carbonarius* [7] while absence of OTA and fumonisin biosynthetic genes and presence of putative secondary metabolite clusters for asperazine and NGP production in *A. tubingensis* G131 [2] have been shown through comparative genome analyses. The knowledge gained through genome sequencing and annotation can be used to construct genome-scale metabolic models (GSMM), which can provide a tool for multi-omics integration as well as for in silico strain design. A number of tools are available for automated construction of draft genome-scale models from genome sequence [8]. These draft models may be capable of producing biomass; however, they generally lack reliability when experimental results are simulated. This makes manual curation a critical step for refining and optimizing a draft model. Due to the unique ability of *A. niger* to produce citric acid in high titres, it was the first *Aspergillus* sp. whose GSMM was reconstructed. Since the first attempt in 2008 [9], several rounds of improvements in the model have been carried out with the most recent model published by [10]. Similar reconstructions have also been carried out for *A. terreus* [11], *A. oryzae* [12] and *A. nidulans* [1]*.* However, no attempt at modelling the metabolism of *A. tubingensis* has been made to date. Availability of a GSMM of *A. tubingensis* would help in understanding the metabolic responses of this organism to various process conditions. Given the ability of *Aspergillus* strains to metabolise both glucose and xylose, the primary sugars in lignocellulosic hydrolysates, it would be of interest to simulate the metabolic response of *A. tubingensis* to these sugars.

In this work, the whole genome of a wild strain *A. tubingensis* DJU120 isolated in this study was de novo sequenced, assembled, annotated, and subsequently used for the reconstruction of a GSMM of *A. tubingensis*, offering a systematic understanding into the organism's metabolism. The predictive capability of the model (*i*MK1652) was validated, comparing constraint-based modelling outcomes with experimentally determined growth rates and growth-phenotype microarray data.

## Methods

### Isolation of A. tubingensis DJU120 strain

The *A. tubingensis* DJU120 wild strain was isolated from a dead *Gladiolus* leaf showing signs of a fungal infection using a direct plating technique with a medium selective for fungi (potato dextrose agar + 100 μg/mL streptomycin). The species was determined by sequencing (LightRun Tube, Eurofins Genomics) a 0.7 kb region of the calmodulin gene amplified by PCR using primers An_calm_fw (5'-TGTGAGTGCTCCCTGAATGAC-3') and An_calm_rv (5'-CGAACTCGTTGTCTGGTAGC-3').

### Genome sequencing and assembly

Mycelia for DNA extraction were grown in complete medium [13] in 100 mL shake flasks at 20 mL working volume. Cultures for DNA extraction were inoculated at 1 × 10^6^ spores/mL and incubated at 30 °C 150 rpm for 16 h. Mycelia were harvested by filtration through a double layer of Miracloth, washed in dH_2_O, and squeeze dried. 100 mg mycelia were transferred to a 2 mL tube to which two 3 mm tungsten carbide beads and 290 μl 50 mM EDTA pH 8 were added. Mycelia were then ground using a TissueLyser set to 30 s^−1^ for 30 s twice, followed by vortexing. 10 μl lyticase (Sigma, L4025) at 10 mg/mL was then added, followed by incubation at 37 °C for 90 min. DNA was then extracted using the Wizard® Genomic DNA purification kit (Promega), according to the manufacturer's instructions. DNA was further purified by ethanol precipitation and resuspended in 5 mM Tris–HCl pH 8.5. DNA was then sent to the University of York Technology Facility for library preparation. The libraries were sequenced at the University of Leeds Next Generation Sequencing Facility using an Illumina HiSeq 3000 platform with 2 × 150 bp sequencing. The sequencing data were assembled into contigs using SPAdes v 3.14.1 [14]. The contigs were filtered to remove contigs with a length below 1 kb or a coverage below 15x.

### Nuclear and mitochondrial genome annotation

The nuclear DJU120 genome was annotated using the fungal version of GeneMark-ES [15]. Taking the *A. tubingensis* mitochondrial genome available in NCBI (NC_007597.1) as reference, the mitochondrial genome for the DJU120 strain was assembled into one complete linear genome and annotated using GeSeq [16].

### GSMM reconstruction

**Fig. 1:**
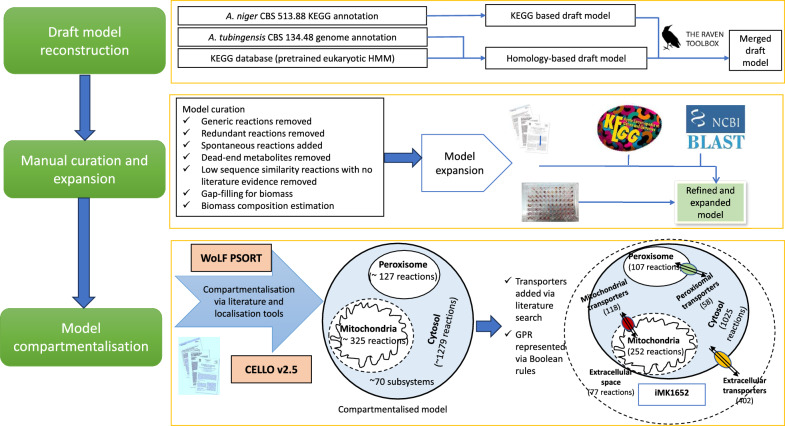
Overview of the steps in the reconstruction of the genome-scale metabolic model of *A. tubingensis*. The process of reconstruction of the genome-scale metabolic model of *A. tubingensis* involved three major steps: draft model reconstruction, manual curation and expansion, and compartmentalisation of the reactions in the model. The draft model reconstruction was based on KEGG annotation of *A. niger* CBS 513.88 as well as *A. tubingensis* annotation to generate a homology-based draft model. The two draft models were then merged. The model was further expanded based on KEGG database, phenotypic microarray data (Biolog plates) and published literature on secondary metabolites. This was followed by compartmentalisation of the reactions using CELLO and WoLF-PSORT, addition of transport reactions and assigning the GPR as well as Boolean rules

The *A. tubingensis* GSMM was reconstructed following three key steps: automated reconstruction procedure, manual curation, and an extensive literature review. Figure 1 gives an outline of the whole reconstruction process.

### Draft model reconstruction using RAVEN toolbox

The protein sequences of *A. tubingensis* CBS 134.48 (annotation, Asptu1) were taken from the JGI database (http://genome.jgi.doe.gov/Asptu1). RAVEN 2.0 toolbox [17] was used for automated draft model reconstruction. The RAVEN 2.0 toolbox contains two approaches for automatic generation of draft models. The first approach relies on the metabolic functions represented in previously published models in KEGG so a draft model using the available *A. niger* CBS 513.88 KEGG annotation was generated. The second approach is based on protein homology where pre-trained sets of eukaryotic HMMs are queried with the protein sequences of *A. tubingensis* CBS 134.48. The draft models generated by both approaches were merged.

### Manual curation of the draft model and addition of secondary metabolite pathways

The initial draft model contained gaps and could not synthesise biomass from glucose. Hence, manual gap filling was initiated to make the model capable of synthesising all the biomass precursors including RNA, DNA, lipid and protein. Initially, non-enzymatic reactions were added to the model to produce the disconnected metabolites. The candidate enzymes were searched in the *A. niger* KEGG database and the homologues were searched in the *A. tubingensis* genome. If no homologous proteins were found, a targeted literature search was carried out. Each pathway in the model was individually checked to remove redundant reactions. The generic reactions were replaced with specific reactions in the model by replacing the generic metabolites (those without exact mass specified) in these reactions with metabolites having a fixed mass. For example, fatty acid metabolism had a number of reactions where the fatty acids were represented by the generic formula R-COOH. These generic fatty acids were replaced with specific fatty acids which have literature evidence based on fatty acid profiles in *Aspergillus* spp. Criteria which specify which protein sequences RAVEN should consider as orthologues were kept as E-value < 1E^−30^, query cover > 200 bp and identity > 40 %. Protein sequences which showed sequence identity below 50 % were manually identified and checked for their presence in *A. tubingensis* based on literature evidence*.* When no literature or homology evidence was found the generic reactions were removed from the model. Similarly, dead-end metabolites were removed if there was no literature evidence on their production in *Aspergillus* spp. Efforts were made to include pathways that correspond to the known degradation capabilities of *A. tubingensis* along with identification of gene clusters associated with secondary metabolite pathways. Such reactions were added on the basis of literature evidence or sequence similarity with well characterized genes in other fungal species.

### Compartmentalisation and transport

The reactions were segregated into three different intracellular compartments (the cytosol, mitochondria and peroxisome) or an extracellular “compartment” based on literature evidence and previous *A. niger* models. WoLF PSORT [18] and CELLO v.2.5 [19] localisation tools were used to confirm the compartmentalisation and identify multi-compartment localised proteins. Reactions without associated genes were compartmentalised according to biochemical and physiological evidence when available. When no supporting literature or associated genes were found, reactions were compartmentalised into the cytosol. Furthermore, an extensive search was carried out to identify the transporters needed to produce all the biomass components. These transporters consist of those involved in uptake, secretion and in inter-organelle exchange such as cytosol-mitochondrial transporters and cytosol-peroxisome transporters. These transporters were added on the basis of sequence homology with functionally-characterised transporters in other fungal species. However, some transporters were also added for the purpose of gap-filling even if no literature support could be found. Lastly, the reactions catalyzed by enzyme complexes and isozymes were identified manually and represented by standard Boolean operators “AND” and “OR”, respectively.

### Biomass composition estimation

#### Organism and cultivation conditions

*A. tubingensis* DJU120 spores were revived on potato dextrose agar slants and allowed to grow for 2–3 days at 37 °C. Spores were harvested using a sterile cotton wool bud and suspended in saline Tween (0.1 % Tween 80, 9 g/L NaCl). Spore suspensions were used to make glycerol stocks and stored at − 80 °C. Spores were counted using a haemocytometer (Rohem, Maharashtra, India) and cultures were inoculated at 1 × 10^6^ spores/mL.

The biomass compositions were measured for the mycelial and pelleted morphologies. The fungus was grown at 30 °C 250 rpm in 30 mL culture media in 250 mL side-baffled shake flasks (Nalgene, Rochester, NY, USA) to obtain filamentous morphology and in 250 mL bottom-baffled flasks, delong neck (Chemglass Life Sciences LLC, USA) to obtain pelleted morphology. To obtain filamentous morphology, the following medium was used: glucose (160 g/L), urea (3.6 g/L), (NH_4_)_2_SO_4_ (0.52 g/L), K_2_HPO_4_ (0.5 g/L), CaCO_3_ (0.03125 g/L), MgSO_4_·7H_2_O (0.275 g/L), ZnSO_4_·7H_2_O (0.00225 g/L), FeSO_4_·7H_2_O (0.0095 g/L), CuSO_4_·5H_2_O (0.0117 g/L), MnCl_2_·(H_2_O)_4_ (0.0000108 g/L), citric acid monohydrate (3.3 g/L), Tween 80 (0.0094%) [20]. The medium was sterilized by autoclaving (121 °C for 15 min), and the pH was adjusted after autoclaving by the addition of sterile H_2_SO_4_. To obtain pelleted morphology, filter sterilised disodium EDTA dihydrate (0.02976 g/L) was added to the medium just before inoculation. After 48 h, the cultures were harvested through a muslin cloth and the harvested biomass was washed three times with deionised water. The washed biomass was collected in pre-weighed 50 mL centrifuge tubes and then frozen overnight at − 80 °C and then lyophilised. The lyophilised biomass was ground in liquid nitrogen and kept overnight at − 80 °C followed by a second round of lyophilisation. The freeze-dried biomass was stored at − 20 °C until the measurement of biomass components. All measurements were done in triplicate.

Citric acid fermentations of glucose-xylose mixture, for validating the model, were conducted in “Hydrolysate Replica” (HR) medium containing glucose (80 g/L), xylose (40 g/L), NaNO_3_ (3 g/L), KH_2_PO_4_ (0.27 g/L), MgSO_4_.7H_2_O (0.71 g/L), FeSO_4_.7H_2_O (0.0151 g/L), ZnSO_4_.7H_2_O (0.0161 g/L), CuSO_4_.5H_2_O (0.0108 g/L), MnSO_4_.H_2_O (0.0046 g/L), Na_2_SO_4_ (2.4 g/L) CaCl_2_ (0.27 g/L) and vegetable peptone (5 g/L). HR medium was developed at York based on analysis of bagasse hydrolysate.

#### Biomass composition estimation

DNA in the freeze-dried biomass samples was measured using the diphenylamine method [21]. 20 mg of powdered biomass was taken in a 2 mL microcentrifuge tube and 1 mL of 50 mM Tris–EDTA (TE) (pH 8.0) buffer was added. This was followed by addition of 150 mg of 0.5 mm glass beads (Sigma). Bead beating was carried out for 20 min (one cycle: 1 min beating followed by 1 min on ice) followed by centrifugation at 4000 g for 10 min. The supernatant was separated and kept on ice. 1 mL of 3 M NaOH was added to the residual biomass, vortexed and boiled for 5 min at 95–100 °C followed by cooling on ice, and a second centrifugation step at 4000 g for 10 min. The supernatant was collected and added to the previous supernatant and kept on ice. The amount of total cellular protein in the combined supernatant was estimated using Pierce BCA Protein Assay Kit (Thermo Fisher Scientific, Cramlington, UK).

Lipid extraction was done using chloroform–methanol procedure according to [22]. 50 mg of powdered biomass was added to a 15 mL glass tube with 2 mL of 15 mM NaCl followed by vortexing and the addition of 6 mL of 2:1 chloroform:methanol. The tube was kept at 30 °C, 200 rpm for 2 h, then centrifuged at 3000 g for 30 min at room temperature. The lower layer was carefully separated and transferred to a pre-weighed 5 mL glass tube. Chloroform was evaporated by flushing with nitrogen gas and the weight of the extracted lipid was measured.

Total cell wall was estimated as described by [23]. 500 mg of powdered biomass was added to a pre-weighed 50 mL centrifuge tube with 25 mL 1 % SDS solution. The tube was kept at 4 °C for 16 h with continuous stirring at 50 rpm. The tube was centrifuged at 7000 g for 10 min at 4 °C. The supernatant was discarded and the pellet was serially washed with 40 mL of 20 %, 40 %, 60 %, 80 % and 100 % ethanol after respective centrifugation steps. The supernatant was discarded and the tube was dried at 50 °C for 16 h. After drying, the weight of the tube was measured and the amount of cell wall was determined.

### Model validation and expansion using growth phenotype results

#### Biolog microplate analysis

The spore suspension was diluted using the FF-IF medium supplied by the manufacturer (Biolog Inc., Hayward, CA) to obtain an initial spore concentration of 1 × 10^4^ spores/mL. 100 μl suspension was dispensed in each well of Biolog FF MicroPlates. Inoculated microplates were incubated at 30 ºC for 5 days, and the plates were read at 12 h intervals using a microplate reader at 750 nm (biomass indicator) and 490 nm (colour change indicator). Two replicates were performed several days apart using different spore suspension. The experimental results were compared with the *in-silico* prediction and modifications were made to the model in the case of any discrepancies.

#### Analytical methods

To monitor changes in biomass dry weight, extracellular metabolites (glucose, xylose, and citric acid), and extracellular phosphate, fermentations were non-destructively sampled by taking 500 µl homogeneous samples in pre-dried, pre-weighed 1.5 mL Eppendorf tubes. Samples were centrifuged at 20,238 g for 3 min. Supernatant was transferred to a new 1.5 mL Eppendorf tube and stored at − 20 °C. Biomass pellets were retained for biomass determination and stored at − 20 °C. To determine the concentrations of glucose, xylose, and citric acid in samples of supernatant, enzymatic assay kits were used (K-GLUC, K-XYLOSE, and K-CITR, respectively (Megazyme International Ireland Ltd., Wicklow, Ireland)). To determine the concentration of phosphate, an assay kit was used (ab65622; Abcam, Cambridge, UK). To determine biomass dry weight, biomass samples retained in pre-dried, pre-weighed 1.5 mL Eppendorf tubes were washed 7 times in 1 mL dH_2_O, followed by drying at 70 °C to constant weight. Biomass samples were centrifuged at 20,238*g* for 3 min between washing steps to pellet the biomass, and the supernatant was removed by pipetting without disturbing the biomass pellet.

### Dynamic modelling using the iMK1652 model with fitting to DJU120 in vivo fermentation data

Dynamic modelling was done as described previously [20] with some modifications. The RNA values in the *i*MK1652 biomass equation were set to those used in iDU1756 [10] as this enabled a closer biomass fit to in vivo data. The NGAM was set to 1.9 mmol gDW^−1^ h^−1^, consistent with previous work [20]. The sequential uptake of glucose and xylose was modelled by disabling xylose transport-mediated uptake at external glucose concentrations above 5 mM. As glucose was present at concentrations above 5 mM throughout the simulation, xylose uptake only occurred via passive means. Low-affinity transport-mediated glucose uptake was enabled above 75 g/L glucose. The kinetic parameters applied in the model are given in Table S1 (Additional file 1). The dFBA start time was adjusted to 10 h after inoculation.

## Results

### Genome sequencing, assembly and annotation

#### Genome sequencing and assembly

A total of 284 contigs with an average length of 123,088 bp were assembled from DJU120 genome sequencing data all with length above 1 kb and coverage above 15x. The longest assembled contig was 1,019,403 bp in length. Combined, the contigs represented 34.96 Mb of sequence, similar in length to the genomes of *A. tubingensis* reported previously [2, 24].

#### Nuclear and mitochondrial genome annotation

An initial network was constructed based on *A. tubingensis* CBS 134.48 genome annotation (Asptu1) and did not include the complete mitochondrial genome. Nine genes involved in the electron transport chain, namely six NADH dehydrogenase subunits (1–6) and three ATP synthase subunits (6–9) were missing. The mitochondrial genome of *A. tubingensis* DJU120 was identified using the mitochondrial genome of *A. tubingensis* 0932 (33,656 bp) [25] as the reference sequence*.* One large contig of 32,481 bp, obtained by whole genome de novo assembly of *A. tubingensis* DJU120 genome sequence showed > 98 % identity with the mitochondrial genome sequence of *A. tubingensis* 0932. The assembled genome was then annotated using GeSeq [16]. The mitochondrial genome of *A. tubingensis* DJU120 contains 43 genes encoding two ribosomal RNAs, 25 transfer RNAs, 14 genes involved in respiratory chain complexes and two ORFs in the intergenic regions (*orf1* and *orf2*). The map of the mitochondrial genome is shown in Fig. S1 (Additional file 1). The genes include cytochrome c oxidase subunits 1–3 (*cox1*, *cox2*, and *cox3*), apocytochrome b (*cob*), NADH dehydrogenase subunits 1–6 (*nad1*, *nad2*, *nad3*, *nad4, nad4L*, *nad5*, and *nad6*) and ATP synthase subunits 6–9 *(atp6*, *atp8*, and *atp9*) and two additional ORFs (unidentified reading frame). Nuclear and mitochondrial genomes were compared to determine if any DNA fragments were transferred between them. No cases of similarity were found indicating no transfer of genes between the mitochondrial and nuclear genomes.

To further expand the reconstructed network, annotated ORFs from the genome sequence of *A. tubingensis* DJU120 were assigned to the model as well (Additional file 2). Of the 1651 ORFs from the CBS 134.48 genome assigned to the network, 1595 had direct candidates in the *A. tubingensis* DJU120 genome sequence (> 90 % identity and E-values < 1E^−20^). Another 29 ORFs had candidates with high similarity (70–90 % identity and E-values below 1E^−20^). 26 genes showed 27–70 % identity or E-value > 1E^−10^ with DJU120 gene assignments; these gene ids were individually checked for the functional domains they encode. Except for two genes, all others were found to encode the same domains as in their CBS 134.48 homologues; these genes were retained in the model (Additional file 2).

### GSMM reconstruction

#### Draft model reconstruction using RAVEN toolbox

The high sequence similarity between *A. niger* and *A. tubingensis* motivated us to create the first draft model using the *A. niger* CBS 513.88 KEGG annotation as a template [2]. The *A. tubingensis* model thus generated contained 1440 reactions and 1075 genes, excluding incomplete reactions and reactions with undefined stoichiometry. However, using *A. niger* KEGG model as a template has the disadvantage that the resulting draft model can only contain genes, and their associated reactions, that have high sequence identity in *A. niger*. To include the function of *A. tubingensis* genes that were not represented in the *A. niger* genome, a second draft model was generated based on protein homology of *A. tubingensis* CBS 134.48 sequences against pre-trained eukaryotic HMMs downloaded from KEGG as part of RAVEN toolbox functionalities. Genes which show E-value < 1E^−30^, query coverage > 200 bp and identity > 40 % against already annotated genes in KEGG were included. The second draft model consisted of 1763 reactions and 1891 genes.

The first draft contained 56 reactions not present in the second draft model. The presence of proteins catalysing these reactions was manually checked in the genome of *A. tubingensis.* Homologous proteins for 48 reactions could be found and these reactions were added to the model along with the corresponding gene ids.

#### Manual curation of the draft model – removal of dead-end metabolites and generic reactions

Because the draft model was unable to simulate biomass production, the first step towards curation of the model was gap-filling with the aim to enable growth. Taking the biomass equation from the *A. niger* model (iJB1325) as a reference for the initial gap filling, reactions were added such that the production of all the biomass precursors was possible. The gap-filled model was able to simulate growth on glucose. To further improve the model, 418 reactions resulting in dead-end metabolites were removed from the model (Additional file 3). Literature was searched for evidence of production of these metabolites in *A. tubingensis*. The subsystems aflatoxin biosynthesis, metabolism of xenobiotics by cytochrome P450, caffeine metabolism and drug metabolism which are disconnected and lacked information regarding their activity in *A. tubingensis* were removed from the model*.* 66 generic reactions (Additional file 3) were also removed and replaced with specific reactions. These reactions consisted mostly of the ones involved in fatty acid elongation/degradation, glycerophospholipid metabolism and sphingolipid metabolism. Reactions which can accept more than one cofactor were analyzed. Literature was searched for enzyme studies detailing the cofactor requirements for these reactions. For instance, L-arabinose reductase and D-xylose reductase were found to be specifically NADPH dependent in filamentous fungi [26, 27]. Thus, the reactions catalyzed by these two enzymes utilizing NADH as cofactor were removed from the model. Similarly, the two methylglyoxal reductases isolated from *A. niger* required NADPH specifically for their activities [28] along with dihydrofolate reductase which utilizes NADH less efficiently compared to NADPH [29]. In cases where the cofactor requirements for the reactions were unknown, reactions involving NADH or NADPH were both included. Certain reactions absent in the *A. niger* KEGG database were added to the model by RAVEN. These reactions were manually checked and removed (≈144) if the identity to the well-characterised genes from other *Aspergillus* spp. was below 50% and no literature evidence was found (Additional file 3). Literature was searched for studies of various *Aspergillus* spp. to get insights on the new individual genes and gene clusters that have been characterized in recent years.

#### Manual curation of the draft model—addition of secondary metabolite pathways

*A. tubingensis* CBS 134.48 genome did not contain the OTA biosynthesis gene cluster. However, genes showing sequence similarity to *A. niger* ochratoxinase enzyme were found. Thus, the OTA degrading reaction was added to the model. The ability of *A. tubingensis* to produce the potent antioxidant pyronanogrin A was reported earlier [30]. This motivated us to search for homologues in the *A. tubingensis* genome using the pyronanogrin A synthesis gene cluster (*pyrA-D*) identified in *P. thymicola* IBT5891 [31]. Homologues to all four genes were found and the reactions corresponding to pyronanogrin A biosynthesis were added to the model. Although other pyronanogrins (E, F, G, H, I, J and K) are reported in *A. niger* [32], the *pyn* gene cluster responsible for the production was found to be absent in *A. tubingensis.* Furthermore*, ada* gene cluster, responsible for the production of TAN-1612 (a neuropeptide Y antagonist), has been identified in *A. niger*. This cluster was also identified in the genome of *A. tubingensis* G131 [2] as well as *A. tubingensis* CBS 134.48 (this study). Recently, the biosynthetic pathway for synthesis of alkylcitric acids including hexylcitric acid, hexylaconitic acid and hexylitaconic acid has been elucidated in *A. niger* [33]*.* The genes showing sequence similarity to five genes* (akcA-D* and *cadA)* characterized by [33] were also identified in the *A. tubingensis* CBS 134.48 genome. As also observed in *A. niger*, four genes (*akcA-D*) are present in a single cluster while the *cadA* gene was found to be localised outside the cluster. Based on this evidence, the reactions for synthesis of alkylcitric acids were also added to the model.

Indole-diterpenes (IDTs) are mycotoxins that cause potent neurotoxic and tremorgenic symptoms in insects and mammals along with being important compounds for future drug discovery. Many of these IDTs are derived from paspaline, an intermediate metabolite. This makes paspaline the founding member of this group of IDTs which have also been reported in *A*. *flavus* and *A. oryzae* [34]*.* In *P. paxillin*, four genes (*paxC*, *paxG*, *paxM* and *paxB*) mediate paspaline biosynthesis [35]*.* A cluster containing homologues to three genes (*paxC, paxM* and *paxB*) was found in *A. tubingensis.* However, the GGPP synthase gene, involved in IDT biosynthesis, was not present in the cluster. It is possible that this gene is substituted by the primary metabolic GGPP synthase gene. Similarly, scattered gene locations were also observed for the specific IDT (terpendole K) gene cluster of *T. album* [34]. Thus, the reactions for paspaline biosynthesis were added to the model. The production of asperpyrone A, asperpyrone D, aurasperone A, aurasperone E, dianhydroaurasperone C [36] as well as malformin A1 [36, 37] has been reported in *A. tubingensis*. Therefore, reactions for synthesis of these metabolites were added to the model. The genome of CBS 134.48 was also searched for clusters corresponding to yanuthone, kotanin, azanigerones and carlosic acid biosynthetic pathway using the known *A. niger* characterized clusters for these metabolites. However, no genes with sufficient sequence similarity were found. Furthermore, on the basis of latest reports, pathways for citramalate biosynthesis [38], glycine betaine biosynthesis [39], *trans*-4-hydroxy-L-pipecolic acid [40] and itaconate degradation [41] were also added to the model while these pathways are missing from the latest *A. niger* models (Table S2, Additional file 1). *Aspergillus* is known to metabolise certain plant derived secondary metabolites such as naringenin, quercetin, phloretin, chlorogenic acid and pectin. Reactions (along with corresponding gene ids) for the uptake and metabolism of these metabolites were also added to the model. Fatty acid synthesis in filamentous fungi including *A. nidulans* and *A. oryzae* is reported to be carried out by Type I fatty acid synthase (FAS) complex. The FAS complex contains all the catalytic domains necessary for synthesis of C16-18 fatty acids. These domains are distributed on two multidomain polypeptide chains (α and β) [42]. A similar enzyme nomenclature was used for fatty acid synthesis reactions in the *A. tubingensis* model while in both the latest *A. niger* models (iJB1325 and iDU1756) bacterial nomenclature was used. Thus, over 40 secondary metabolism pathways were added to the model based on recent and past literature (Table S2, Additional file 1).

### Compartmentalisation of the reactions and addition of transport reactions

**Fig. 2:**
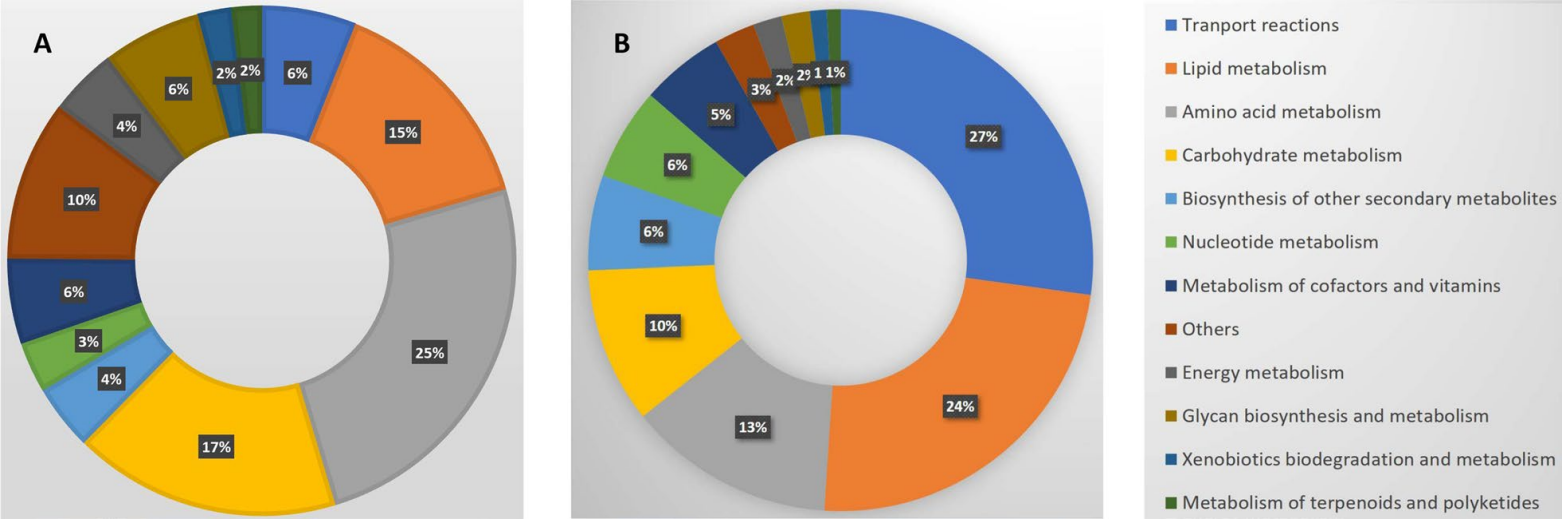
ORF (**A**) and reaction distribution (**B**) in various subsystems in the model

*i*MK1652 accounts for four compartments (extracellular, cytosol, mitochondria and peroxisome). This generation of a fully compartmentalised model required addition of intercompartmental transport reactions. The model consists of 1652 ORFs out of a total of 11,368 ORFs (for DJU120). A total of 309 extracellular transporters, 102 mitochondrial transporters and 50 peroxisomal transporters were added to the model. Certain novel plasma membrane transporters, which were absent in the latest *A. niger* models, were added to the model on the basis of recent literature. For instance, manganese [43], citrate [44] and cellodextrin [45] transporter reactions with corresponding genes were added. Mitochondrial shuttle proteins which transport citric acid from mitochondria to the cytosol in exchange for cytosolic organic acid are important to citric acid production in *Aspergillus* spp*.* Earlier models include a mitochondrial citrate-malate shuttle (*ctpA*) while in the current model another potential transporter *cocA* [46] which is a mitochondrial citrate-oxoglutarate shuttle protein was also added. In total, 61 enzyme complexes were included in the network. A comparison of our model with the latest *A. niger* models, *i*JB1325 [47] and *i*DU1756 [10], is shown in Table 1. The model has a total of 2039 reactions. The distribution of the ORFs and the reactions in the model into twelve different subsystems based on KEGG pathway database is provided in Fig. 2. Subsystems that have a high fraction of complexes had a larger fraction of ORFs compared to reactions, and the subsystems which do not have annotated genes assigned in the model had a greater fraction of reactions. For example, transport reactions had the largest fraction of reactions (27 %, Fig. 2B). However, many of the transport reactions do not have a corresponding ORF. Therefore, the ORF fraction of the transport reactions was only 6 % (Fig. 2A). A large fraction of the ORFs correspond to amino acid metabolism (25 %), lipid metabolism (15 %) and carbohydrate metabolism (17 %), which were also the most represented fractions in the reactions at 13 %, 24 %, and 10 %, respectively.

**Table 1:** Comparison of *A. tubingensis* model with the recently published *A. niger* models

	*A. niger* model (*i*JB1325, 2018)	*A. niger* model (*i*DU1756, 2020)	*A. tubingensis* model (*i*MK1652, this study)
*Reactions*			
Total	2320	1862	2039
Extracellular transport reactions + extracellular reactions	762	475	470
Reactions taking place in the cytosol + transport reactions	1229	1011	1415
Reactions taking place in the mitochondria + transport reactions	313	247	372
Reactions taking place in the peroxisome + transport reactions	163	121	151
*Metabolites*			
Total	1818	1309	1631
Extracellular metabolites	420	232	270
Metabolites present in cytosol	1001	803	957
Metabolites present in mitochondria	253	183	303
Metabolites present in peroxisome	138	95	120
*Genes*			
Total	1325	1756	1652

### Biomass composition estimation

*A. tubingensis* DJU120 was cultivated under different conditions to obtain two different morphologies: filamentous and pelleted. The biomass compositions in these different morphologies were measured. Interestingly, most measured macromolecules showed similar levels in the two morphologies, with the biggest differences in the lipid levels followed by the cell wall (Table 2).

**Table 2:** Biomass composition of *A. tubingensis* in filamentous and pelleted morphology and comparison with *A. niger* biomass composition (all values in g/g DW)

Biomass component	*A. tubingensis*, Filamentous morphology (this study)	*A. tubingensis*, Pelleted morphology (this study)	*A. niger* biomass composition [9, 47]	*A. niger* biomass composition [10]
DNA	0.0022 ± 0.0001	0.0028 ± 0.0002	0.0024	0.00081
Protein	0.1279 ± 0.0110	0.1424 ± 0.017	0.263	0.263
Lipid	0.20 ± 0.02	0.11 ± 0.01	0.108	–
Cell wall	0.43 ± 0.02	0.48 ± 0.03	0.38	0.38
RNA	0.00604*	0.00604*	0.018	0.00604
Ash	0.0750*	0.0750*	0.0750	-
Pool	0.1310*	0.1310*	0.1310	-
TOTAL	0.9685 ± 0.051	0.9481 ± 0.047	0.9774	-

*Taken from [10] for RNA values and [47] for others

### Model validation and expansion using growth phenotype results

The substrate utilizing ability of *A. tubingensis* DJU120 was analyzed using a Biolog assay [48]. Out of the 95 carbon sources tested, 56 were found to enable growth while 39 did not. The uptake reactions for 13 of the 56 carbon sources that enabled growth were missing from the model (Additional file 4). The metabolic pathways for three of these (gentiobiose, maltotriose, and palatinose) are known and so were added to the model. Also, there were certain carbon sources (D-galactose, D-arabinose, maltose, D-ribose and lactose) that the organism could not use as sole carbon sources. However, the genes encoding the pathways for the metabolism of these were found in the genome. These metabolic pathways were, therefore, retained in the model. Furthermore, we compared the substrate utilisation pattern of the wild-type *A. niger* ATCC 1015 [47] strain with *A. tubingensis* DJU120 strain by comparing their respective Biolog data. Because the plates used by [47] are different from the FF plates used in the current study, we could only compare the pattern for 48 carbon sources. Out of these 48 carbon sources, 14 did not show similar utilisation patterns (Additional file 4). This indicates some key differences in the substrate utilisation of these two *Aspergillus* species.

### Dynamic modelling using the iMK1652 model with fitting to DJU120 in vivo fermentation data

**Fig. 3:**
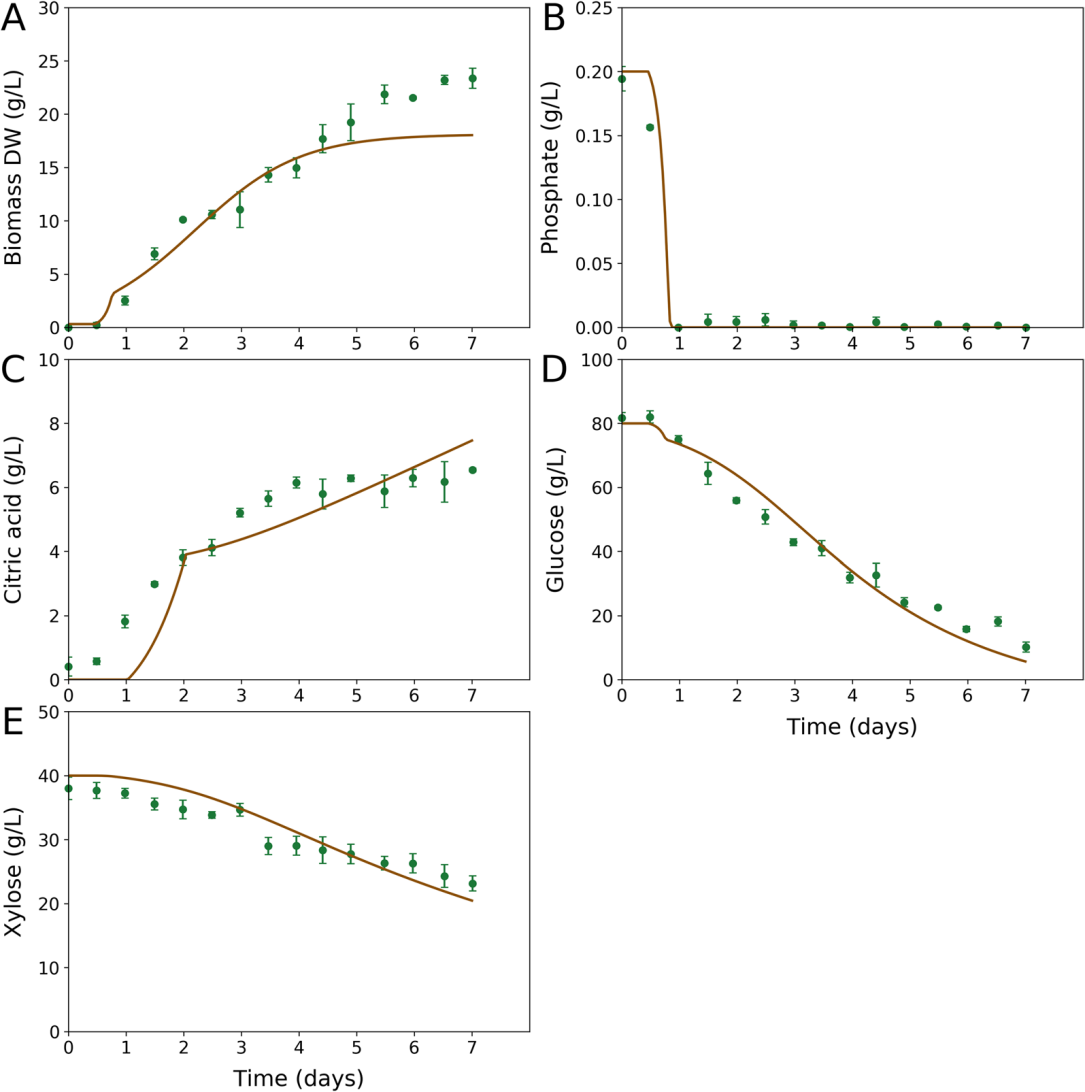
Model-predicted vs. actual fermentation of a glucose-xylose mixture by *A. tubingensis*. Dynamic FBA was conducted using the reconstructed genome-scale metabolic model to simulate the sequential uptake of glucose and xylose. The solid lines represent the simulation results from the dFBA run for biomass (**A**), phosphate (**B**), citric acid (**C**), glucose (**D**) and xylose (**E**). Experimentally, *A. tubingensis* DJU120 strain was used to ferment a mixture of glucose (80 g/L) and xylose (40 g/L). The dots and the associated error bars represent the experimental data ± s.d. from (n = 3) different runs

The measured composition of the biomass under pelleted morphology (which is relevant for citric acid production) was used to create the biomass equation (see Additional file 5 for the complete model in Excel format and Additional file 6 for the model in SBML format). The coefficient of RNA in the biomass equation was the same as that used in [10]. The *i*MK1652 model was applied in a dynamic modelling framework [20] to evaluate its response to a mixed C-source fermentation. The model closely captured DJU120 fermentative data on glucose, xylose, and phosphate consumption, as well as biomass and citric acid production (Fig. 3), showing its applicability to capture citric acid fermentation of lignocellulosic biomass hydrolysate.

## Discussion

In this work, we have reconstructed a GSMM of *A. tubingensis* that contains secondary metabolism pathways and models the fermentation of mixed sugars well. Several aspects of the model were manually curated to gap-fill the pathways for the synthesis of individual biomass precursors, including recently identified pathways and transporters. A significant effort was made to investigate the inclusion of the secondary metabolism pathways. These include pathways for citramalate biosynthesis [38], glycine betaine biosynthesis [39], trans-4-hydroxy-L-pipecolic acid [40] and itaconate degradation [41]. Additionally, based on recent identifications, several transporters were added to the model, such as those for manganese [43], citrate [44] and cellodextrin [45] as well as mitochondrial citrate-oxoglutarate shuttle transporter [46]. The presence of the associated genes/proteins was manually checked in the genome of *A. tubingensis*.

A major advantage of *A. tubingensis* over *A. niger* is that it does not secrete toxins. Indeed, several mycotoxin synthesis pathways were either absent or incomplete in the genome, providing a genomic basis for the non-toxinogenic nature of this species [2]. Interestingly, the pathway for synthesis of OTA was absent while the enzyme for degradation of OTA was present in the genome of *A. tubingensis*. This is consistent with a previous study which reported the *A. tubingensis* strains M036 and M074 exhibited OTA-biodegradation activity but not OTA-producing activity [6]. This suggests that *A. tubingensis* could be a potential candidate for bioremediation of OTA. Understanding of these biosynthetic pathways is vital for genetic engineering. Thus, including these pathways in the genome-scale model becomes imperative if the model has to be explored for broader applications. Pathways for the synthesis of other secondary metabolites found in *A. niger* but not in *A. tubingensis* included Pyronanogrin, Ada, Vanuthone, Azanigerones, and carlosic acid. Thus, the differences in the secondary metabolism of *A. niger* and *A. tubingensis* were comprehensively covered in the present model.

Pathways of certain metabolites such as the subsystems aflatoxin biosynthesis, metabolism of xenobiotics by cytochrome P450, caffeine metabolism and drug metabolism were removed from the model because there were gaps in the pathways and information regarding their activity in *A. tubingensis* is lacking*.* Many of these pathways have not been annotated in KEGG and thus required extensive literature review. As more information becomes available on the pathways of metabolism of these compounds and the associated genes are identified, these pathways can be added to the model in future.

In this study, we determined the biomass composition of *A. tubingensi*s to inform the biomass equation in the model and subsequently create a model that more accurately reflects the actual biomass composition. In doing so, we observed key discrepancies with *A. niger* models in protein and cell wall content. The protein content of *A. tubingensis* biomass was about half of that in *A. niger* models while the cell wall content was higher. Also, the lipid and cell wall contents were found to vary with changes in morphology. This is in agreement with other studies that have shown that lipid metabolites [49] and cell wall modulation [50] affect fungal morphology, and stresses the importance of basing models on actual biomass composition data for the target organism and conditions, rather than assuming the same biomass composition as existing models for similar organisms.

Currently, the model does not include the essential metabolites such as folate, biotin and thiamine, etc. in the biomass equation. It, however, provides pathway for synthesis of these metabolites. The focus of the present work was on macromolecules (protein, nucleic acid, lipid, cell wall) which contribute significantly to the biomass dry weight. The stated small molecules, even though they may be essential, don't contribute significantly to the biomass dry weight and were excluded in the model for sake of simplicity. Future studies could do more comprehensive biomass composition analyses to further improve the model.

## Conclusions

Our work is the first reported genome-scale metabolic model of the non-toxinogenic citric acid producing fungus *A. tubingensis*. All the major metabolic pathways as well as many secondary metabolism pathways were included in the model after extensive manual curation. The model closely fits the experimental data for growth and product formation on a mixture of sugars. This model will serve as an important framework for systems biology studies of *A. tubingensis*.

### Supplementary Information

**Additional file 1.** Table of kinetic parameters used in the dFBA (Table S1); Table of a pathway-based comparison of *A. tubingensis* genome-scale metabolic model with two published models of *A. niger* (Table S2), and a figure of the annotation of the mitochondrial genome of DJU120 (Figure S1).

**Additional file 2.** An Excel file showing a genomic comparison of *A. tubingensis* CBS 134.48 (annotation, Asptu1) with *A. tubingensis* DJU120, model gene IDs and distribution of model gene IDs as highly aligned, mid aligned and low aligned in the two genomes.

**Additional file 3.** An Excel file showing the reactions removed from the draft model due to low sequence similarity, generation of dead-end metabolites, absence of cofactors, generic reactions, no literature evidence or repetition.

**Additional file 4.** An Excel file containing the phenotypic microarray (Biolog plate) data for DJU120, a list of new C-sources added to the model based on the data, and phenotypic differences between *A. tubingensis* DJU120 and *A. niger* on the Biolog plates.

**Additional file 5.** An Excel file of the model containing model compartments, reactions, genes and metabolites.

**Additional file 6.** The model in SBML format.

## Data Availability

The datasets used and/or analyzed during the current study are available from the corresponding author upon reasonable request.

## References

[1] David H, Özçelik IŞ, Hofmann G, Nielsen J (2008). Analysis of *Aspergillus nidulans* metabolism at the genome-scale. BMC Genomics.

[2] Choque E, Klopp C, Valiere S, Raynal J, Mathieu F (2018). Whole-genome sequencing of *Aspergillus tubingensis* G131 and overview of its secondary metabolism potential. BMC Genomics.

[3] Frisvad JC, Larsen TO, Thrane U, Meijer M, Varga J, Samson RA (2011). Fumonisin and ochratoxin production in industrial *Aspergillus niger* strains. PLoS ONE.

[4] Carboué Q, Maresca M, Herbette G, Roussos S, Hamrouni R, Bombarda I (2020). Naphtho-gamma-pyrones produced by *Aspergillus tubingensis* G131: new source of natural nontoxic antioxidants. Biomolecules.

[5] Khan S, Nadir S, Shah ZU, Shah AA, Karunarathna SC, Xu J (2017). Biodegradation of polyester polyurethane by *Aspergillus tubingensis*. Environ Pollut.

[6] Cho SM, Jeong SE, Lee KR, Sudhani HPK, Kim M, Hong SY (2016). Biodegradation of ochratoxin A by *Aspergillus tubingensis* isolated from meju. J Microbiol Biotechnol.

[7] Cabañes FJ, Sanseverino W, Castellá G, Bragulat MR, Cigliano RA, Sánchez A (2015). Rapid genome resequencing of an atoxigenic strain of *Aspergillus carbonarius*. Sci Rep.

[8] Machado D, Andrejev S, Tramontano M, Patil KR (2018). Fast automated reconstruction of genome-scale metabolic models for microbial species and communities. Nucleic Acids Res.

[9] Andersen MR, Nielsen ML, Nielsen J (2008). Metabolic model integration of the bibliome, genome, metabolome and reactome of *Aspergillus niger*. Mol Syst Biol.

[10] Upton DJ, McQueen-Mason SJ, Wood AJ (2020). In silico evolution of Aspergillus Niger organic acid production suggests strategies for switching acid output. Biotechnol Biofuels.

[11] Liu J, Gao Q, Xu N, Liu L (2013). Genome-scale reconstruction and in silico analysis of *Aspergillus terreus* metabolism. Mol Biosyst.

[12] Vongsangnak W, Olsen P, Hansen K, Krogsgaard S, Nielsen J (2008). Improved annotation through genome-scale metabolic modeling of *Aspergillus oryzae*. BMC Genomics.

[13] Arentshorst M, Ram AFJ, Meyer V (2012). Using non-homologous end-joining-deficient strains for functional gene analyses in filamentous fungi. Methods Mol Biol.

[14] Nurk S, Bankevich A, Antipov D, Gurevich A, Korobeynikov A, Lapidus A, et al. Assembling genomes and mini-metagenomes from highly chimeric reads. In: Deng, M., Jiang, R., Sun, F., Zhang, X. (eds) Research in Computational Molecular Biology. RECOMB 2013. Lecture Notes in Computer Science vol 7821:158–170.

[15] Ter-Hovhannisyan V, Lomsadze A, Chernoff YO, Borodovsky M (2008). Gene prediction in novel fungal genomes using an ab initio algorithm with unsupervised training. Genome Res.

[16] Tillich M, Lehwark P, Pellizzer T, Ulbricht-Jones ES, Fischer A, Bock R (2017). GeSeq—Versatile and accurate annotation of organelle genomes. Nucleic Acids Res.

[17] Wang H, Marcišauskas S, Sánchez BJ, Domenzain I, Hermansson D, Agren R (2018). RAVEN 2.0: a versatile toolbox for metabolic network reconstruction and a case study on *Streptomyces coelicolor*. PLoS Comput Biol.

[18] Horton P, Park KJ, Obayashi T, Fujita N, Harada H, Adams-Collier CJ (2007). WoLF PSORT: Protein localization predictor. Nucleic Acids Res.

[19] Yu CS, Chen YC, Lu CH, Hwang JK (2006). Prediction of protein subcellular localization. Proteins.

[20] Upton DJ, McQueen-Mason SJ, Wood AJ (2017). An accurate description of *Aspergillus niger* organic acid batch fermentation through dynamic metabolic modelling. Biotechnol Biofuels.

[21] Herbert D, Phipps PJ, Strange RE (1971). Chemical analysis of microbial cells. Methods Microbiol.

[22] Bligh EG, Dyer WJ (1959). A rapid method of total lipid extraction and purification. Can J Biochem Physiol.

[23] Mahadevan PR, Tatum EL (1965). Relationship of the major constituents of the *Neurospora crassa* cell wall to wild-type and colonial morphology. J Bacteriol.

[24] Yoshioka I, Takahashi H, Kusuya Y, Yaguchi T, Kirimura K (2020). Draft genome sequence of *Aspergillus tubingensis* WU-2223L, a citric acid-producing filamentous fungus belonging to Aspergillus Section Nigri. Microbiol Resour Announc.

[25] Juhász Á, Engi H, Pfeiffer I, Kucsera J, Vágvölgyi C, Hamari Z (2007). Interpretation of mtDNA RFLP variability among *Aspergillus tubingensis* isolates. Antonie Van Leeuwenhoek.

[26] Mojzita D, Penttilä M, Richard P (2010). Identification of an L-arabinose reductase gene in *Aspergillus niger* and its role in L-arabinose catabolism. J Biol Chem.

[27] Mojzita D, Vuoristo K, Koivistoinen OM, Penttilä M, Richard P (2010). The, “true” l-xylulose reductase of filamentous fungi identified in *Aspergillus niger*. FEBS Lett.

[28] Inoue Y, Rhee H, Watanabe K, Muraka K, Kimura A (1988). Metabolism of 2-oxoaldehyde in mold: Purification and characterization of two methylglyoxal reductases from *Aspergillus niger*. Eur J Biochem.

[29] Schweitzer BI, Dicker AP, Bertino JR (1990). Dihydrofolate reductase as a therapeutic target. FASEB J.

[30] Samson RA, Houbraken JAMP, Kuijpers AFA, Frank JM, Frisvad JC (2004). New ochratoxin A or sclerotium producing species in Aspergillus section Nigri. Stud Mycol.

[31] Tang MC, Zou Y, Yee D, Tang Y (2018). Identification of the pyranonigrin A biosynthetic gene cluster by genome mining in *Penicillium thymicola* IBT 5891. AIChE J.

[32] Awakawa T, Yang XL, Wakimoto T, Abe I (2013). Pyranonigrin E: A PKS-NRPS hybrid metabolite from *Aspergillus niger* identified by genome mining. ChemBioChem.

[33] Palys S, Pham TTM, Tsang A (2020). Biosynthesis of alkylcitric acids in *Aspergillus niger* involves both co-localized and unlinked genes. Front Microbiol.

[34] Kozák L, Szilágyi Z, Tóth L, Pócsi I, Molnár I (2019). Tremorgenic and neurotoxic paspaline-derived indole-diterpenes: biosynthetic diversity, threats and applications. Appl Microbiol Biotechnol.

[35] Saikia S, Parker EJ, Koulman A, Scott B (2007). Defining paxilline biosynthesis in *Penicillium paxilli*: functional characterization of two cytochrome P450 monooxygenases. J Biol Chem.

[36] Zhan J, Gunaherath GMKB, Wijeratne EMK, Gunatilaka AAL (2007). Asperpyrone D and other metabolites of the plant-associated fungal strain *Aspergillus tubingensis*. Phytochemistry.

[37] Tan QW, Gao FL, Wang FR, Chen QJ (2015). Anti-TMV activity of malformin A1, a cyclic penta-peptide produced by an endophytic fungus *Aspergillus tubingensis* FJBJ11. Int J Mol Sci.

[38] Hossain AH, Hendrikx A, Punt PJ (2019). Identification of novel citramalate biosynthesis pathways in *Aspergillus niger*. Fungal Biol Biotechnol.

[39] Lambou K, Pennati A, Valsecchi I, Tada R, Sherman S, Sato H (2013). Pathway of glycine betaine biosynthesis in *Aspergillus fumigatus*. Eukaryot Cell.

[40] Hibi M, Mori R, Miyake R, Kawabata H, Kozono S, Takahashi S (2016). Novel enzyme family found in filamentous fungi catalyzing trans-4-hydroxylation of L-pipecolic acid. Appl Environ Microbiol.

[41] Chen M, Huang X, Zhong C, Li J, Lu X (2016). Identification of an itaconic acid degrading pathway in itaconic acid producing *Aspergillus terreus*. Appl Microbiol Biotechnol.

[42] Tamano K, Bruno KS, Karagiosis SA, Culley DE, Deng S, Collett JR (2013). Increased production of fatty acids and triglycerides in *Aspergillus oryzae* by enhancing expressions of fatty acid synthesis-related genes. Appl Microbiol Biotechnol.

[43] Fejes B, Fejes B, Ouedraogo JP, Fekete E, Sándor E, Flipphi M (2020). The effects of external Mn2+concentration on hyphal morphology and citric acid production are mediated primarily by the NRAMP-family transporter DmtA in *Aspergillus niger*. Microb Cell Fact.

[44] Steiger MG, Rassinger A, Mattanovich D, Sauer M (2019). Engineering of the citrate exporter protein enables high citric acid production in *Aspergillus niger*. Metab Eng.

[45] Lin H, Zhao J, Zhang Q, Cui S, Fan Z, Chen H (2020). Identification and characterization of a cellodextrin transporter in *Aspergillus niger*. Front Microbiol.

[46] Kirimura K, Kobayashi K, Yoshioka I (2019). Decrease of citric acid produced by *Aspergillus niger* through disruption of the gene encoding a putative mitochondrial citrate-oxoglutarate shuttle protein. Biosci Biotechnol Biochem.

[47] Brandl J, Aguilar-pontes MV, Schäpe P, Noerregaard A, Arvas M, Ram AFJ (2018). A community-driven reconstruction of the *Aspergillus niger* metabolic network. Fungal Biol Biotechnol.

[48] Hobbie EA, Watrud LS, Maggard S, Shiroyama T, Rygiewicz PT (2003). Carbohydrate use and assimilation by litter and soil fungi assessed by carbon isotopes and BIOLOG® assays. Soil Biol Biochem.

[49] Gao J, Liu H, Zhang Z, Liang Z (2023). Quorum sensing-mediated lipid oxidation further regulating the environmental adaptability of *Aspergillus ochraceus*. Metabolites.

[50] Kisser M, Kubicek CP, Röhr M (1980). Influence of manganese on morphology and cell wall composition of *Aspergillus niger* during citric acid fermentation. Arch Microbiol.

