# Supplementary material for: Reconstruction of a genome-scale metabolic model and in-silico flux analysis of Aspergillus tubingensis: a non-mycotoxinogenic citric acid-producing fungus
Source: Biotechnol Biofuels Bioprod. 2024 May 28;17:70. doi: 10.1186/s13068-024-02506-4 (PMC11134751; doi:10.1186/s13068-024-02506-4)
Supplement: Supplementary file 1 — Additional file 1. Table of kinetic parameters used in the dFBA (Table S1); Table of a pathway-based comparison of A. tubingensis genome-scale metabolic model with two published models of A. niger (Table S2), and a figure of the annotation of the mitochondrial genome of DJU120 (Figure S1). [file 13068_2024_2506_MOESM1_ESM.docx]

**Additional file 1**

**Table S1. Kinetic parameters applied in dynamic modelling.**

| **Parameter** | **Description** | **Value** |
| --- | --- | --- |
| $v_{Pe,max}$ (mmol gDW^-1^ h^-1^) | External phosphate maximum input rate | 0.15 |
| $K_{Pe}$ (mM) | External phosphate Michaelis constant | 0.0333^a^ |
| $v_{P,max}$ (mmol gDW^-1^ h^-1^) | Internal phosphate maximum input rate | 0.12 |
| $K_{P}$ (mM) | Internal phosphate Michaelis constant | 46.67 |
| $v_{G1}$ (mmol gDW^-1^ h^-1^) | External glucose passive uptake rate | 0.0009 × [GLC]^b^ |
| $v_{G2,max}$ (mmol gDW^-1^ h^-1^) | External glucose high-affinity transport-mediated uptake maximum rate | 0.04 |
| $K_{G2}$ (mM) | External glucose high-affinity transport-mediated uptake Michaelis constant | 0.26^a^ |
| $K_{i2}$ (mM) | External glucose high-affinity transport-mediated uptake citrate inhibition constant | 933^a^ |
| $v_{G3,max}$ (mmol gDW^-1^ h^-1^) | External glucose low-affinity transport-mediated uptake maximum rate | 2.706^a^ |
| $K_{G3}$ (mM) | External glucose low-affinity transport-mediated uptake Michaelis constant | 3.67^a^ |
| $K_{i3}$ (mM) | External glucose low-affinity transport-mediated uptake citrate inhibition constant | 233.21^a^ |
| $v_{X1}$ (mmol gDW^-1^ h^-1^) | External xylose passive uptake rate | 0.00033 × [XYL]^c^ |
| $v_{CIT}$ (mmol gDW^-1^ h^-1^) | Citric acid output rate constraint^d^ | 0.01 |

^a^These values are the same as used in Upton *et al.,* 2017

^b^[GLC] is the concentration of external glucose in mM

^c^[XYL] is the concentration of external xylose in mM

^d^Citric acid output rate constraint was only applied 38 hours after the start time

**Table S2.** **Pathway-based comparison of *A. tubingensis* genome-scale metabolic model with two published models of *A. niger*.**

| **Pathway** | ***A. tubingensis (This study)*** | ***A. niger* (iJB1325)** (Brandl et al. 2018) | ***A. niger* (iDU1756)** (Upton et al., 2020) |
| --- | --- | --- | --- |
| 1. Glycine betaine biosynthetic pathway | Added. Its production is reported in *A. fumigatus* and *ChoA* and *Badh* characterised (Lambou et al., 2013) | Not added | Not added |
| 1. Ochratoxin A biosynthetic pathway | Not added (No homologue found) | Added | Added |
| 1. OTA hydrolysis | Added (homologue found; degradation reported in Cho et al., 2016) | Added | Not added |
| 1. Histamine synthesis pathway | Not added, no literature evidence found for histidine decarboxylase (E.C no 4.1.1.22) in *Aspergillus* spp. | Added, protein id added refers to different E.C. no. 4.1.1.28 than expected | Added |
| 1. Paspaline biosynthesis pathway (*atmG*, *atmC*, *atmM* and *atmB*) | Added. These four genes are sufficient to mediate paspaline biosynthesis in *P. paxillin* (**Saikia et al., 2007***).* Paspaline-derived IDTs reported in *A*. *flavus* and *A. oryzae (*Kozak et al., 2019) | Not added | Only *atmG* gene added |
| 1. *trans*-4-hydroxy-l-pipecolic acid biosynthesis pathway | Added (are valuable building blocks for the organic synthesis of pharmaceuticals) (Hibi et al., 2016) | Not added | Not added |
| 1. Itaconate biosynthetic pathway | Added (Hossain et al., 2016) | Not added | Added |
| 1. Biodegradation pathway for itaconate | Added (Chen et al., 2016) | Not added | Not added |
| 1. Citramalate biosynthesis and transport reaction | Added (Hossain et al., 2019) | Not added | Not added |
| 1. Indolepyruvic acid (IPA) biosynthetic pathway | Added (Sardar et al., 2018) | Not added | Added |
| 1. E.C. no. and corresponding protein ids for cytosolic fatty acid synthesis pathway | Added based on yeast nomenclature | Uses bacterial enzyme nomenclature | Uses bacterial enzyme nomenclature |
| 1. Fumonisin B2, B4 and B6 metabolites | No such metabolite added | Added to metabolite list, however, no reactions consuming or producing any of the above metabolite added in the model | No such metabolite added |
| 1. Manganese transporter | Added (Fejes et al., 2020) | Reaction added but no gene | No reaction added |
| 1. Citrate transporter | Added (Steiger et al., 2019) | Not added | Not added |
| 1. Itaconate transporter | Added (Hossain et al., 2016) | Reaction not added | Reaction added with no gene |
| 1. Mitochondrial citrate-oxoglutarate shuttle reaction | Reaction with gene added (Kirimura et al., 2019) | Not added | Not added |
| 1. Malformin biosynthetic pathway | Only reactions for synthesis of Malformin A1 added (Tan et al., 2015) | Reactions for synthesis of Malformin A1, A2, A4, B1A, B2, B3, B4, B5 and C added | No reaction added |
| 1. Asperpyrone biosynthetic pathway | Only metabolites asperpyrone A and D added (Zhan et al., 2007) | Reactions for synthesis for Asperpyrone B and C added | No reaction added |
| 1. T4HN (1,3,6,8-Tetrahydroxynaphthalene) biosynthetic pathway | Added (Chiang et al., 2010) | Added | Not added |
| 1. Aurasperone biosynthetic pathway | Aurasperone A, B and E added (Zhan et al., 2007) | Aurasperone A, B and C added | Not added |
| 1. Dianhydroaurasperone biosynthetic pathway | Dianhydroaurasperone C (Zhan et al., 2007) metabolite added | Not added | Not added |
| 1. Nigragillin biosynthetic pathway | Not added. No literature on production in *A. tubingensis* available | Nigragillin synthesis reaction added. Nigerazine A and B metabolites added | Not added |
| 1. Arsenite efflux pump (*acrA*) | Added (Choe et al., 2012) | Added | Not added |
| 1. Arsenate reductase | Not added. No literature or homologue found in *Aspergillus* spp. | Added | Not added |
| 1. 6-methylsalicylate (6-MSA) biosynthetic pathway | No homologue found in *A. tubingensis* | Added | Added |
| 1. Yanuthone biosynthetic pathway | Not added. Homologues not found for *yan* cluster in *A. tubingensis* | Biosynthesis reactions for Yanuthone D, E, X1,X2, M,L,K,J,F,G,H and I added | Not added |
| 1. Tyrosine O-prenyltransferase reactions [catalyzing prenylations of both tyrosine and tryptophan derivatives](https://idp.springer.com/authorize/casa?redirect_uri=https://link.springer.com/article/10.1007/s00253-014-5872-7&casa_token=OTx570OfyroAAAAA:ydOL7M8wELjGKo343W3EuVNBU41awc5PI68YAweUTcmAdest8WJwj6iK5qRzbGG2ytGZWkgIxTew7XGO) showing the broad substrate range | Added Fan et al., 2014) | Added | Not added |
| 1. Kotanin biosynthesis pathway | Not added. Biosynthetic cluster not present in *A. tubingensis.* Homologues not found (Girol et al., 2012) | Added | Not added |
| 1. Pyranonigrin E,F,G,H,I,J and K biosynthesis pathway | Biosynthetic cluster not present in *A. tubingensis.* Homologues not found (Awakawa, et al., 2013) | Pyranonigrin E,F,G,H,I,J and K biosynthesis pathway added | Not added |
| 1. Pyranonigrin A biosynthesis pathway | Biosynthetic cluster present in *A. tubingensis.* (Tang et al., 2018). Pyranonigrin A reported in *A. tubingensis* (Samson et al., 2004) | Pyranonigrin A metabolite added but no synthesis reaction added | Not added |
| 1. Azanigerones E biosynthesis pathway | Not added. No homologues found *(*Zabala et al., 2012*).* | Azanigerones A–F biosynthesis pathway added | Not added |
| 1. TAN-1612 biosynthesis pathway | Added (Li et al., 2011). Biosynthetic cluster also reported in *A. tubingensis* G131 ([Choque](https://link.springer.com/article/10.1186/s12864-018-4574-4#auth-1) et al., 2018) | Added | Not added |
| 1. Type III PKS reactions for the synthesis of triketide and tetraketide pyrones | Added (Li et al., 2011) | Added | Not added |
| 1. Carlosic acid biosynthesis pathway | Not added. Gene cluster not found (Yang et al., 2014) | Carlosic acid biosynthesis pathway added | Not added |
| 1. Alkylcitric acid biosynthesis pathway | Added (Palys et al., 2019) | Not added | Not added |
| 1. An alternative protocatechuic acid metabolic pathway through hydroxyquinol | Added (Lubbers et al., 2019) | Not added | Not added |
| 1. Phenol degradation pathway | Added through KEGG database. Phenol degradation has been reported for *A. niger* and *A. fumigatus* | Not added | Added |
| 1. Cellodextrin transporter and metabolism pathway | Added (Lin et al., 2020) | Transporter and metabolic pathway not added | Transport reaction added with no gene |
| 1. 3-Guanidinopropanoate catabolism pathway | Added (Saragadam et al., 2019) | Not added | Not added |
| 1. Aminoacyl-tRNA biosynthesis pathway | Added (Datt et al., 2014) | Not added | Added |
| 1. N-Glycan biosynthesis pathway | Added (KEGG) | Not added | Not added |
| 1. Glycosylphosphatidylinositol (GPI)-anchor biosynthesis pathway | Added (KEGG) | Not added | Not added |
| 1. NAD dependent lactate dehydrogenase (LDH) | Not added. Since no *Aspergillus* spp. known to produce lactic acid and neither a functional LDH was reported. Dave et al (2015) characterised a putative *ldhA* gene from *A.* *niger* and it was found not to code for a functional LDH | Added | Added |

Figure S1: *A. tubingensis* DJU120 mitochondrial genome annotation


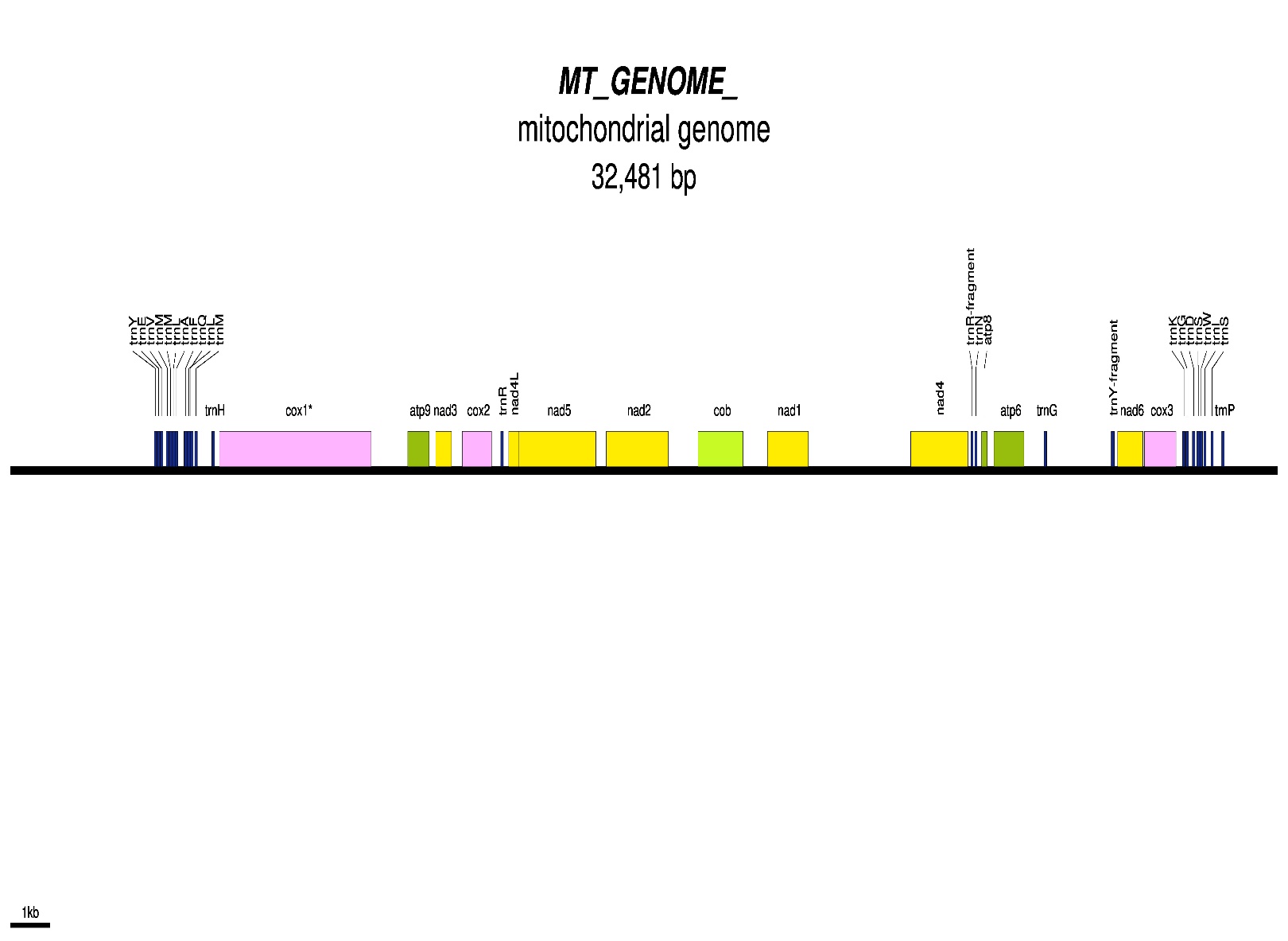


**References**

1. Lambou, K., Pennati, A., Valsecchi, I., Tada, R., Sherman, S., Sato, H., Beau, R., Gadda, G. and Latgé, J.P., 2013. Pathway of glycine betaine biosynthesis in Aspergillus fumigatus. *Eukaryotic cell*, *12*(6), pp.853-863.
2. Cho, S.M., Jeong, S.E., Lee, K.R., Sudhani, H.P., Kim, M., Hong, S.Y. and Chung, S.H., 2016. Biodegradation of Ochratoxin a by Aspergillus tubingensis isolated from Meju. *J Microbiol Biotechnol*, *26*(10), pp.1687-1695.
3. Tan, Q.W., Gao, F.L., Wang, F.R. and Chen, Q.J., 2015. Anti-TMV activity of malformin A1, a cyclic penta-peptide produced by an endophytic fungus Aspergillus tubingensis FJBJ11. *International journal of molecular sciences*, *16*(3), pp.5750-5761.
4. Saikia, S., Parker, E.J., Koulman, A. and Scott, B., 2007. Defining paxilline biosynthesis in Penicillium paxilli functional characterization of two cytochrome P450 monooxygenases. *Journal of Biological Chemistry*, *282*(23), pp.16829-16837.
5. Kozák, L., Szilágyi, Z., Tóth, L., Pócsi, I. and Molnár, I., 2019. Tremorgenic and neurotoxic paspaline-derived indole-diterpenes: biosynthetic diversity, threats and applications. *Applied microbiology and biotechnology*, *103*(4), pp.1599-1616.
6. Hibi, M., Mori, R., Miyake, R., Kawabata, H., Kozono, S., Takahashi, S. and Ogawa, J., 2016. Novel enzyme family found in filamentous fungi catalyzing trans-4-hydroxylation of l-pipecolic acid. *Appl. Environ. Microbiol.*, *82*(7), pp.2070-2077.
7. Hossain, A.H., Li, A., Brickwedde, A., Wilms, L., Caspers, M., Overkamp, K. and Punt, P.J., 2016. Rewiring a secondary metabolite pathway towards itaconic acid production in Aspergillus niger. *Microbial cell factories*, *15*(1), p.130.
8. Chen, M., Huang, X., Zhong, C., Li, J. and Lu, X., 2016. Identification of an itaconic acid degrading pathway in itaconic acid producing Aspergillus terreus. *Applied microbiology and biotechnology*, *100*(17), pp.7541-7548.
9. Hossain, A.H., Hendrikx, A. and Punt, P.J., 2019. Identification of novel citramalate biosynthesis pathways in Aspergillus niger. *Fungal Biology and Biotechnology*, *6*(1), p.19.
10. Sardar, P. and Kempken, F., 2018. Characterization of indole-3-pyruvic acid pathway-mediated biosynthesis of auxin in Neurospora crassa. *PloS one*, *13*(2).
11. Fejes, B., Ouedraogo, J.P., Fekete, E., Sándor, E., Flipphi, M., Soós, Á., Molnár, Á.P., Kovács, B., Kubicek, C.P., Tsang, A. and Karaffa, L., 2020. The effects of external Mn 2+ concentration on hyphal morphology and citric acid production are mediated primarily by the NRAMP-family transporter DmtA in Aspergillus niger. *Microbial cell factories*, *19*(1), p.17.
12. Steiger, M.G., Rassinger, A., Mattanovich, D. and Sauer, M., 2019. Engineering of the citrate exporter protein enables high citric acid production in Aspergillus niger. *Metabolic engineering*, *52*, pp.224-231.
13. Kirimura, K., Kobayashi, K. and Yoshioka, I., 2019. Decrease of citric acid produced by Aspergillus niger through disruption of the gene encoding a putative mitochondrial citrate-oxoglutarate shuttle protein. *Bioscience, biotechnology, and biochemistry*, *83*(8), pp.1538-1546.
14. Tan, Q.W., Gao, F.L., Wang, F.R. and Chen, Q.J., 2015. Anti-TMV activity of malformin A1, a cyclic penta-peptide produced by an endophytic fungus Aspergillus tubingensis FJBJ11. *International journal of molecular sciences*, *16*(3), pp.5750-5761.
15. Zhan, J., Gunaherath, G.K.B., Wijeratne, E.K. and Gunatilaka, A.L., 2007. Asperpyrone D and other metabolites of the plant-associated fungal strain Aspergillus tubingensis. *Phytochemistry*, *68*(3), pp.368-372.
16. Chiang, Y.M., Oakley, B.R., Keller, N.P. and Wang, C.C., 2010. Unraveling polyketide synthesis in members of the genus Aspergillus. *Applied microbiology and biotechnology*, *86*(6), pp.1719-1736.
17. Choe, S.I., Gravelat, F.N., Al Abdallah, Q., Lee, M.J., Gibbs, B.F. and Sheppard, D.C., 2012. Role of Aspergillus niger acrA in arsenic resistance and its use as the basis for an arsenic biosensor. *Appl. Environ. Microbiol.*, *78*(11), pp.3855-3863.
18. Fan, A., Chen, H., Wu, R., Xu, H. and Li, S.M., 2014. A new member of the DMATS superfamily from Aspergillus niger catalyzes prenylations of both tyrosine and tryptophan derivatives. *Applied microbiology and biotechnology*, *98*(24), pp.10119-10129.
19. Gil Girol, C., Fisch, K.M., Heinekamp, T., Günther, S., Hüttel, W., Piel, J., Brakhage, A.A. and Müller, M., 2012. Regio‐and stereoselective oxidative phenol coupling in Aspergillus niger. *Angewandte Chemie International Edition*, *51*(39), pp.9788-9791.
20. Awakawa, T., Yang, X.L., Wakimoto, T. and Abe, I., 2013. Pyranonigrin E: a PKS‐NRPS hybrid metabolite from Aspergillus niger identified by genome mining. *ChemBioChem*, *14*(16), pp.2095-2099.
21. Tang, M.C., Zou, Y., Yee, D. and Tang, Y., 2018. Identification of the pyranonigrin A biosynthetic gene cluster by genome mining in Penicillium thymicola IBT 5891. *AIChE Journal*, *64*(12), pp.4182-4186.
22. Samson, R.A., Houbraken, J.A.M.P., Kuijpers, A.F., Frank, J.M. and Frisvad, J.C., 2004. New ochratoxin A or sclerotium producing species in Aspergillus section Nigri. *Studies in mycology*, *50*(1), pp.45-56.
23. Zabala, A.O., Xu, W., Chooi, Y.H. and Tang, Y., 2012. Characterization of a silent azaphilone gene cluster from Aspergillus niger ATCC 1015 reveals a hydroxylation-mediated pyran-ring formation. *Chemistry & biology*, *19*(8), pp.1049-1059.
24. Li, Y., Chooi, Y.H., Sheng, Y., Valentine, J.S. and Tang, Y., 2011. Comparative characterization of fungal anthracenone and naphthacenedione biosynthetic pathways reveals an α-hydroxylation-dependent Claisen-like cyclization catalyzed by a dimanganese thioesterase. *Journal of the American Chemical Society*, *133*(39), pp.15773-15785.
25. Choque, E., Klopp, C., Valiere, S., Raynal, J. and Mathieu, F., 2018. Whole-genome sequencing of Aspergillus tubingensis G131 and overview of its secondary metabolism potential. *BMC genomics*, *19*(1), p.200.
26. Li, J., Luo, Y., Lee, J.K. and Zhao, H., 2011. Cloning and characterization of a type III polyketide synthase from Aspergillus niger. *Bioorganic & medicinal chemistry letters*, *21*(20), pp.6085-6089.
27. Yang, X.L., Awakawa, T., Wakimoto, T. and Abe, I., 2014. Three acyltetronic acid derivatives: noncanonical cryptic polyketides from Aspergillus niger identified by genome mining. *ChemBioChem*, *15*(11), pp.1578-1583.
28. Palys, S., Pham, T.T.M. and Tsang, A., 2019. Biosynthesis of alkylcitric acids in Aspergillus niger involves both co-localized and unlinked genes. *bioRxiv*, p.714071.
29. Lubbers, R.J., Dilokpimol, A., Peng, M., Visser, J., Makela, M.R., Hildén, K.S. and de Vries, R.P., 2019. Discovery of Novel p-Hydroxybenzoate-m-hydroxylase, Protocatechuate 3, 4 Ring-Cleavage Dioxygenase, and Hydroxyquinol 1, 2 Ring-Cleavage Dioxygenase from the Filamentous Fungus Aspergillus niger. *ACS Sustainable Chemistry & Engineering*, *7*(23), pp.19081-19089.
30. Lin, H., Zhao, J., Zhang, Q., Cui, S., Fan, Z., Chen, H. and Tian, C., 2020. Identification and Characterization of a Cellodextrin Transporter in Aspergillus niger. *Frontiers in Microbiology*, *11*, p.145.
31. Saragadam, T., Kumar, S. and Punekar, N.S., 2019. Characterization of 4-guanidinobutyrase from Aspergillus niger. *Microbiology*, *165*(4), pp.396-410.
32. Datt, M. and Sharma, A., 2014. Novel and unique domains in aminoacyl-tRNA synthetases from human fungal pathogens Aspergillus niger, Candida albicans and Cryptococcus neoformans. *BMC genomics*, *15*(1), p.1069.
33. Dave, K.K. and Punekar, N.S., 2015. Expression of lactate dehydrogenase in aspergillus Niger for l-lactic acid production. *PloS one*, *10*(12).
